# miR-495 suppresses osteosarcoma growth and metastasis by directly targeting RUNX3 in the PI3K/Akt pathway *in vitro* and *in vivo*

**DOI:** 10.3389/fcell.2026.1647277

**Published:** 2026-03-13

**Authors:** Rongkai Shen, Meng Chen, Xia Zhu, Jianhua Lin

**Affiliations:** 1 Department of Orthopedics, The First Affiliated Hospital of Fujian Medical University, Fuzhou, Fujian, China; 2 Department of Orthopedics, National Regional Medical Center, Binhai Campus of the First Affiliated Hospital, Fujian Medical University, Fuzhou, China

**Keywords:** metastasis, miR-495, osteosarcoma, PI3K/Akt signaling pathway, RUNX3

## Abstract

**Objective:**

This study aimed to identify miR-495 as a potential regulator in osteosarcoma and characterize its role in tumor progression and underlying signaling pathways.

**Methods:**

Differentially expressed miRNAs in osteosarcoma were screened from the GSE39058 dataset using bioinformatics analysis. miR-495 expression was validated in tissues/cells using S-MED database and qPCR. Functional assays, including proliferation, invasion, and apoptosis analyses, were performed following transfection with miR-495 mimics or inhibitors. Transcriptome sequencing combined with dual-luciferase assays identified RUNX3 as a direct target, and Western blot analyzed PI3K/Akt pathway activation.

**Results:**

miR-495 was downregulated in osteosarcoma tissues/cells, positively correlating with patient survival. miR-495 overexpression inhibited cell proliferation, invasion, and migration, while promoting apoptosis by suppressing PI3K/Akt pathway (downregulating p-Akt, Bcl-2; upregulating Bax, c-casp3). RUNX3 overexpression rescued these effects, confirming it as a functional target.

**Conclusion:**

miR-495 acts as a tumor suppressor in osteosarcoma by targeting RUNX3 to inhibit PI3K/Akt signaling, suggesting its potential as a prognostic marker and therapeutic target.

## Introduction

Osteosarcoma is the most common primary malignant bone tumor, predominantly affecting adolescents and young adults. Research suggests that it stems from mesenchymal stem cells, marked by the atypical bone formation by neoplastic cells (Author Anonymous, 2022). Despite more than three decades of intensive research, therapeutic strategies and clinical outcomes for osteosarcoma have shown limited improvement. Osteosarcoma can arise in any bone but preferentially involves the metaphyseal regions of long bones. The distal femur is the most frequently affected site, followed by the proximal tibia and proximal humerus, whereas other anatomical locations account for less than 10% of cases (Cole et al., 2022). Upon diagnosis, approximately 10%–20% of individuals exhibit metastatic disease, most commonly involving the lungs, and the 5-year survival rate for these patients remains below 20%–30% (Zhang et al., 2023).

miR-495 has been found to exhibit diverse functions in various types of cancer. Studies in non-small cell lung cancer (Formosa et al., 2014), papillary thyroid carcinoma (Alves and Geraldo, 2023), breast cancer (Liu et al., 2022) and bladder cancer (Zhou et al., 2021) have identified miR-495 as a tumor suppressor. In contrast, in several malignant solid tumors, an increased expression of miR-495 is associated with tumor progression (Chen et al., 2013; Hwang-Verslues et al., 2011). The role of miR-495 in osteosarcoma, however, is seldom reported, necessitating further in-depth investigation.

The RUNX3 gene, part of the runt-related transcription factor family, maps to the p36-35 region on chromosome 1 in humans. Its tissue-restricted expression contributes to its dualistic role as either a tumor suppressor or an oncogene across a spectrum of human malignancies. The silencing of RUNX3 is linked to the genesis of diverse cancer types, and the genomic locus 1p36-35, housing the RUNX3 gene, is frequently subject to deletions in a variety of cancers. Such deletions are observed in neoplastic samples from the colon, urinary bladder, mammary gland, lungs, stomach, liver, and neuroblastic tumors (Nomoto et al., 2000; Ezaki et al., 1996; Schwab et al., 1996; Matsumoto et al., 2004). Hypermethylation of the RUNX3 promoter has been identified as a major risk factor for bladder cancer, with individuals exhibiting high levels of RUNX3 methylation showing an approximately 100-fold increased risk (Kim et al., 2005; Kandimalla et al., 2013). Literature suggests that RUNX3 inhibits the proliferation and invasion of breast cancer (Wang N. et al., 2024). In contrast, elevated RUNX3 levels in osteosarcoma and epithelial ovarian cancer imply a potential oncogenic function in these malignancies (Otani et al., 2022; Lee et al., 2011). Moreover, RUNX3 expression positively correlates with tumor aggressiveness in head and neck squamous cell carcinoma (Tsunematsu et al., 2009).

Recurrence and metastasis remain major challenges in osteosarcoma management and represent a principal reason for the stagnation in patient survival over the past three decades (Averill, 2021). This study aimed to clarify the expression pattern and biological function of miR-495 in osteosarcoma, and further explored whether miR-495 regulates osteosarcoma progression through direct targeting of RUNX3 and modulation of the PI3K/Akt signaling pathway, both *in vitro* and *in vivo*.

## Materials and methods

### Patients’ data

Sixty paired osteosarcoma tissue samples and adjacent non-tumorous tissues were archived in the sample repository of Fujian Orthopedic Research Institute, with pathological confirmation. All cases underwent systematic follow-up, during which patient survival outcomes were meticulously documented. This study was granted ethical approval by the Institutional Ethics Committee (Ethics Approval Number: MRCTA, ECFAH of FMU [2021] 156), and all participants provided written informed consent after full disclosure of study objectives and procedures.

## Bioinformatics analysis and screening of osteosarcoma-associated miRNAs

The GEO database (Gene Expression Omnibus database, https://www.ncbi.nlm.nih.gov/geo/) was queried using the terms “osteosarcoma,” “recurrence,” and “metastasis” to identify relevant GSE (Gene Expression Omnibus Series) datasets associated with osteosarcoma metastasis and recurrence. Gene names were converted using R language and the GPL15762 platform annotation file. Differential miRNAs were screened using the limma package in R (Ritchie et al., 2015), with criteria set as |log2 FC| > 0.58 and FDR (adjusted p-value) <0.05.

### KEGG pathway enrichment analysis of miRNAs

To explore the roles of differentially expressed miRNAs in KEGG pathways, the differential miRNAs were uploaded into the online analysis tool DIANA TOOLS mirpathe (http://diana.imis.athena-innovation.gr/DianaTools/index.php), and miR-495 with high functional pathway enrichment was screened out.

### Expression differences of miRNAs across diverse tissues

The miRNAs were uploaded into the Sarcoma-microRNA Expression Database (S-MED, https://www.oncomir.umn.edu/SMED/index.php) to analyze the expression profiles of miR-495 in normal tissues and various sarcoma subtypes.

Subsequently, the osteosarcoma tissues from 60 patients were stratified into high- and low-expression groups based on miR-495 expression levels. Survival analysis was performed by integrating patient survival time and status, and Kaplan-Meier curves were plotted to visualize the results.

### Cell culture

The osteoblastic cell line hFOB 1.19 and osteosarcoma cell lines MG63, U2OS, 143B, HOS, and SaOS2 were obtained from the Cell Bank of the Chinese Academy of Sciences Typical Culture Collection Committee (Shanghai, China). The culture conditions were as follows:

hFOB 1.19 cells were maintained in DMEM/F12 medium (Gibco, Carlsbad, CA, United States) supplemented with 0.3 mg/mL G418 (Gibco, Carlsbad, CA, United States), 10% fetal bovine serum (FBS, Gibco, Carlsbad, CA, United States), 100 mg/mL penicillin (Gibco, Carlsbad, CA, United States), and 100 mg/mL streptomycin (Gibco, Carlsbad, CA, United States).

MG63, U2OS, 143B, and HOS cells were cultured in DMEM containing 10% FBS (Gibco, Carlsbad, CA, United States), 100 U/mL penicillin, and 100 mg/mL streptomycin.

SaOS2 cells were grown in RPMI 1640 medium (Gibco, Carlsbad, CA, United States) supplemented with 10% FBS, 100 mg/mL penicillin, and 100 mg/mL streptomycin.

All cell lines were cultured at 37 °C in a humidified atmosphere with 5% CO_2_.

### Cell transfection

The miR-495 mimic, miR-495 negative control (NC), miR-495 inhibitor, and miR-495 inhibitor NC (inhibitor NC) were synthesized by Shanghai GenePharma Co., Ltd. (Shanghai, China) (Table 1). Cell transfection was performed using Lipofectamine 3000 (Invitrogen, Carlsbad, CA, United States) according to the manufacturer’s instructions.

**TABLE 1:** Sequences of miR-495 Mimic and Inhibitor.

Name	Sequences (5′–3′)
mimic	AAA​CAA​ACA​UGG​UGC​ACU​UCU​UGA​AGU​GCA​CCA​UGU​UUG​UUU​UU
mimic NC Sense	UUC​UCC​GAA​GUU​GUC​ACG​UTT
NC Antisense	ACG​UGA​CAC​GUU​CGG​AGA​ATT
inhibitor	AAG​AAG​UGC​ACC​AUG​UUU​GUU​U
inhibitor NC	GAG​UAC​UUU​UGU​GUA​GUA​CAA

### Quantitative real-time PCR (qRT-PCR)

Total RNA was extracted from osteosarcoma cells using TRIzol reagent (Invitrogen), and reverse-transcribed into cDNA with a reverse transcription kit from Tiangen Biotech (Beijing, China). Quantitative detection was performed using the Tiangen real-time fluorescence quantitative qPCR kit (Beijing, China) and the miRcute Enhanced miRNA Fluorescence Quantitative Detection Kit (Beijing, China) according to the manufacturer’s instructions. U6 served as an internal reference. Primers for miR-495, U6, and RUNX3 were synthesized by Sangon Biotech Co., Ltd (Shanghai, China) (Tables 1, 2). The relative expression levels of RUNX3 and miR-495 were calculated using the 2^−ΔΔCt^ method.

**TABLE 2:** Primer Sequences for miR-495, RUNX3, and U6.

Name	Sequences (5′–3′)
miR-495 F	GCG​AAA​CAA​ACA​TGG​TGC​A
miR-495 R	AGT​GCA​GGG​TCC​GAG​GTA​TT
RUNX3 F	CAA​CTT​CCT​CTG​CTC​CGT​GCT​G
RUNX3 R	TTC​TCG​TCA​TTG​CCT​GCC​ATC​AC
U6 F	CTTCGGCAGCACATATAC
U6 R	GAA​CGC​TTC​ACG​AAT​TTG​C

### Cell proliferation, colony formation, invasion, and wound healing assays

#### Cell proliferation assay

Cell proliferation was detected using the CCK-8 Kit (APExBIO, Shanghai, China) according to the manufacturer’s protocol. Cells were seeded into 96-well plates at a density of 2.0 × 10^3^ cells/well. At 24-, 48-, 72-, and 96-h post-seeding, the optical density (OD) at 450 nm was measured using an automated microplate reader (Bio-Rad Laboratories, United States). Each sample was assayed in triplicate, and cell growth curves were plotted.

#### Colony formation assay

Osteosarcoma 143B and HOS cells were seeded into 6-well plates at 1.0 × 10^3^ cells/well and cultured for 14 days with medium changes every 2 days. Colonies were fixed with 4% paraformaldehyde, stained with 0.5% crystal violet solution (Sigma, Germany), scanned for imaging, and colonies containing >50 cells were counted. Each sample was replicated three times.

#### Transwell invasion assay

Transwell chambers with 8-μm pores coated with Matrigel (Corning Incorporated, Corning, NY, United States) were prepared according to the manufacturer’s instructions to assess changes in the invasive capacity of 143B and HOS cells. Detailed procedures followed a previously published method (Justus et al., 2023). Each sample was tested in triplicate.

#### Wound healing assay

Cells were seeded into 24-well plates at 1.5 × 105 cells per well. After 24 h, a sterile 1-mL pipette tip was used to create a scratch along the diameter of each well. Detached cells were washed away, and 500 μL of DMEM medium without fetal bovine serum was added to each well. Cells were incubated at 37 °C in a 5% CO_2_ atmosphere. Images were captured immediately (0 h) and 24 h after scratching to calculate the migration rate. Each group included three replicate wells.

#### Cell apoptosis assay

The osteosarcoma 143B and HOS cells transfected with miR-495 mimic or NC were collected and processed according to the instructions of the FITC Annexin V Apoptosis Detection Kit I (BD, Franklin Lakes, NJ, United States). The apoptosis rate was measured using a FACScan® flow cytometer (BD, United States). The results were presented as scatter plots, with the upper right and lower right quadrants representing the proportions of late apoptotic and early apoptotic cells, respectively. The total apoptosis rate was calculated as the sum of early and late apoptotic cells.

### Establishment of miR-495 stably expressed cell lines

The miR-495 stable transfection plasmid and its control plasmid miR-495 NC (Normal Control) were purchased from Shanghai Genechem Co., Ltd. (Shanghai, China). The miR-495 overexpression plasmid was constructed on the GV514 vector backbone (CMV-EGFP-MCS-SV40-Neomycin), with the target sequence inserted at the XhoI/BamHI cloning sites. The corresponding negative control plasmid was Catalog No. CON107, with no exogenous insert sequence. After transfecting osteosarcoma 143B cells with Lipofectamine 3000 (Invitrogen) for 48 h, the transfection efficiency was observed under a fluorescence inverted microscope. The cells were then screened and expanded in DMEM complete medium containing 0.8 mg/mL G418 (Promega, Madison, WI, United States). RT-PCR was used to verify the changes in miR-495 expression levels in the stably transfected 143B osteosarcoma cell line. The 143B cells stably transfected with the miR-495 plasmid were named 143B-495 cells, while those transfected with miR-495 NC were named 143B-NC cells.

### Transcriptome sequencing of osteosarcoma cells

The osteosarcoma 143B-495 and 143B-NC cells were routinely cultured in 10-cm culture dishes. When the cell confluence reached 80%–90%, the culture medium was discarded, and the cells were washed twice with PBS. After removing the PBS completely, 1 mL of Trizol Reagent was added to each dish. Three biological replicates were set for each group. The total RNA was then sent to Shanghai Geneuine Biotechnology Co., Ltd. for stranded transcriptome sequencing. Transcriptome sequencing was performed on an Illumina NovaSeq 6000 platform (Illumina, San Diego, CA, United States) with paired-end 150 bp reads. Differentially expressed genes (DEGs) were identified using DESeq2 with a threshold of |log_2_FC(Fold Change)| ≥ 1 and adjusted *p*-value <0.05. The RNA sequencing data generated in this study have been deposited in the National Genomics Data Center (NGDC) database under accession number PRJCA058457.

### Prediction of miR-495 target genes

The potential target genes of miR-495 were predicted by querying the miRNA target prediction databases miRDB, TarBase, TargetScan, and miRTarBase with miR-495.

Differentially downregulated genes identified from transcriptome sequencing of osteosarcoma 143B-495 and 143B-NC cells were cross-referenced with the bioinformatics-predicted target genes from miRDB, TarBase, TargetScan, and miRTarBase. The intersection of these gene sets was further selected to obtain the preliminary identified target genes.

### Dual-luciferase reporter assay

The wild-type (RUNX3 3′UTR) and mutant (RUNX3 3′UTR mut) sequences of the RUNX3 mRNA 3′-untranslated region (3′UTR) were designed based on the complementary sequences between miR-495 and RUNX3. The gene fragments were amplified by PCR, digested with restriction enzymes, and ligated into the GV272 vector. 293T cells were transfected using Lipofectamine 3000 (Invitrogen) and divided into six groups:

① miR-495 + GV272 CON,
② miR-495 NC + GV272 CON,
③ miR-495 + RUNX3 3′UTR,
④ miR-495 NC + RUNX3 3′UTR,
⑤ miR-495 + RUNX3 3′UTR mut,
⑥ miR-495 NC + RUNX3 3′UTR mut.

Relative luciferase activity was measured using the Dual-Luciferase® Reporter Assay System E1910 (Promega, Madison, WI, United States) according to the manufacturer’s instructions.

### miR-495 modulates osteosarcoma cell functions via RUNX3

Osteosarcoma 143B cells were transfected with miR-495 NC, mimic, mimic + CON, or mimic + RUNX3, and designated as four groups: ① NC, ② mimic, ③ mimic + CON, ④ mimic + RUNX3.

Total RNA and protein were extracted 48 h post-transfection for subsequent analyses. miR-495 expression levels were quantified by reverse transcription and qRT-PCR. RUNX3 protein levels were detected by Western blotting.

Forty-eight hours after transfection, the following assays were performed to assess changes in proliferation, colony formation, invasion, migration, and apoptosis: CCK-8 assay for cell proliferation, Colony formation assay, Transwell invasion assay, Wound healing assay for cell migration, Flow cytometry for apoptosis analysis.

### Subcutaneous xenograft tumors and lung metastasis in osteosarcoma

Osteosarcoma 143B-495 and 143B-NC cells in logarithmic growth phase were digested with trypsin, centrifuged, and collected.

For subcutaneous xenograft models: Ten 6–8-week-old SPF-grade BALB/c-nu mice were randomly divided into two groups (n = 5 per group). Cells (2.0 × 10^6^ in 100 μL PBS) were subcutaneously injected into the dorsal region above the forelimb. Tumor length and width were measured twice weekly using calipers, and tumor volume was calculated using the formula: V = (length × width^2^)/2. Mice were sacrificed after 8 weeks, and tumors were excised for weighing and further analysis.

For lung metastasis models: Under aseptic conditions (75% ethanol disinfection), 2.0 × 10^6^ cells in 100 μL PBS were injected into the tail vein of each mouse (n = 5 per group). Four weeks post-injection, mice were euthanized, and lungs were harvested, fixed in 4% paraformaldehyde, and processed for H&E staining to assess metastatic nodules.

### Western blot (WB) analysis

Total protein was extracted from osteosarcoma tissues and cells using RIPA Lysis Buffer (Beyotime, China). Samples were lysed on a horizontal shaker at 30 rpm for 30 min at 4 °C, sonicated, and centrifuged at 12,000 rpm for 10 min at 4 °C. The supernatant was collected, and protein concentration was determined using a BCA Protein Assay Kit (Beyotime, China).

Protein samples (50 μg) were separated by SDS-PAGE and transferred to PVDF membranes at 400 mA for 60 min at 4 °C.

Membranes were blocked with 5% non-fat milk for 60 min and incubated overnight at 4 °C with primary antibodies against RUNX3 (1:1000), PI3K (1:1000), Akt (1:1000), p-Akt (1:1000), Bax (1:1000), Bcl-2 (1:1000), c-casp3 (1:1000), c-casp9 (1:1000), and GAPDH (1:1000) (Cell Signaling Technology, Beverly, MA, United States).

After washing, membranes were incubated with HRP-conjugated secondary antibodies (1:50,000, CST) for 60 min at room temperature in the dark.

Protein bands were visualized using ECL Chemiluminescent Substrate (Beyotime, China) and imaged with a FlourChem Imaging System (United States). Densitometric analysis was performed using ImageJ software (NIH, Bethesda, MD).

### Statistical analysis

Data was analyzed using SPSS 25.0 software (SPSS Inc., Chicago, IL, United States). Measurement data are presented as mean ± standard deviation (SD). Prior to parametric testing, data normality was assessed using the Shapiro–Wilk test, and homogeneity of variances was evaluated using Levene’s test. For comparisons between two groups, Student’s t-test was applied when assumptions were satisfied. For comparisons involving more than two groups, one-way analysis of variance (ANOVA) was performed, followed by Tukey’s *post hoc* test when variances were homogeneous. When variance heterogeneity was detected, Welch’s ANOVA with Games-Howell *post hoc* correction was used. For RNA sequencing analysis, differentially expressed genes were identified using DESeq2, and multiple testing correction was applied using the Benjamini–Hochberg false discovery rate (FDR) method. A *p*-value <0.05 was considered statistically significant.

## Result

### Screening and bioinformatics analysis of miR-495

**FIGURE 1:**
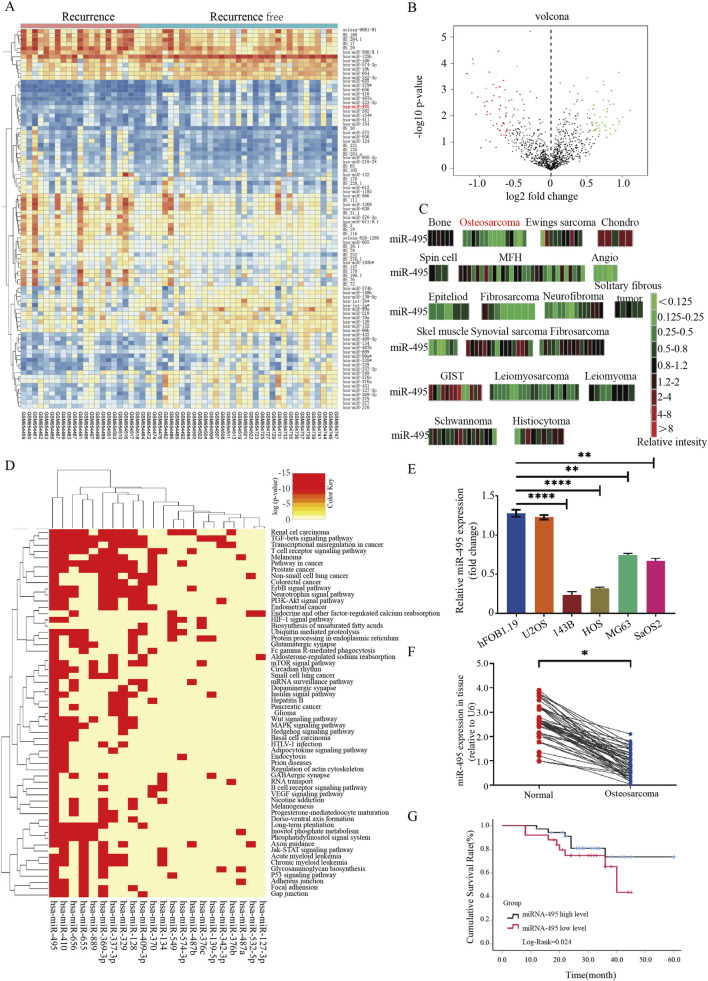
Bioinformatics analysis and screening of osteosarcoma-related miRNAs. **(A)** Heatmap of differentially expressed miRNAs related to osteosarcoma based on analysis of the GSE39058 dataset, 47 miRNAs were upregulated and 43 miRNAs were downregulated. The screening criteria were |log2FC| > 0.58 and FDR <0.05. **(B)** Volcano plot of differentially expressed miRNAs related to osteosarcoma based on analysis of the GSE39058 dataset. **(C)** miR-495 was submitted to the Sarcoma-microRNA Expression Database (S-MED) database for tissue-specific expression profiling and differential analysis across various tissues. **(D)** KEGG pathway enrichment analysis of differentially expressed microRNAs using DIANA TOOLS mirPath, with the heatmap indicating significantly enriched cancer-related pathways. **(E)** Quantitative real-time PCR (qRT-PCR) was performed to compare the expression levels of miR-495 between osteoblasts (hFOB1.19) and osteosarcoma cell lines (U2OS, MG63, SaOS2, HOS, and 143B). The descending order of miR-495 expression was as follows: hFOB1.19 > U2OS > MG63 > SaOS2 > HOS > 143B. **(F)** Quantitative real-time PCR (qRT-PCR) was employed to compare miR-495 expression between 60 paired osteosarcoma tissues and adjacent normal tissues. miR-495 levels were significantly lower in osteosarcoma tissues than in paired adjacent normal tissues. **(G)** Kaplan–Meier survival analysis of osteosarcoma patients stratified into high- and low-miR-495 expression groups (n = 60). In the low miR-495 expression group, the median survival time was 42.22 months (95% CI: 32.38–52.06).In the high miR-495 expression group, the median survival time was 54.22 months (95% CI: 49.01–59.43), with a Log-Rank test value of 0.024.

Differential expression analysis of miRNA data from recurrent and non-recurrent osteosarcoma samples in the GSE39058 dataset identified 43 downregulated and 47 upregulated miRs (Figures 1A,B; Tables 3, 4). Analysis using the S-MED database (https://www.oncomir.umn.edu/SMED/) revealed that miR-495 expression was significantly lower in osteosarcoma tissues than in normal bone tissues (Figure 1C; Table 5). Functional pathway enrichment analysis of differentially expressed miRNAs was performed using the DIANA TOOLS miRPath online tool (http://diana.imis.athena-innovation.gr/DianaTools/index.php). Heatmap visualization showed that miR-495 was significantly enriched in cancer-related functional pathways (Figure 1D).

**TABLE 3:** Downregulated miRNAs in the recurrence-free group vs. the recurrence group.

NO	miRNA	Log2FC	NO	miRNA	Log2FC
1	HS_170	−1.108	23	HS_78	−0.738
2	HS_76	−1.006	24	HS_105	−0.734
3	HS_111	−0.965	25	HS_65	−0.733
4	HS_228.1	−0.958	26	HS_176	−0.715
5	HS_19	−0.949	27	HS_29	−0.691
6	HS_72	−0.948	28	HS_31.1	−0.685
7	hsa-miR-885-3p	−0.932	29	hsa-miR-124	−0.685
8	hsa-miR-638	−0.904	30	hsa-miR-576-3p	−0.685
9	hsa-miR-572	−0.870	31	HS_196.1	−0.677
10	hsa-miR-1268	−0.867	32	HS_283_a	−0.652
11	Solexa-826-1,288	−0.847	33	HS_50	−0.648
12	hsa-miR-122	−0.834	34	hsa-miR-193b*	−0.640
13	hsa-miR-612	−0.830	35	hsa-miR-218-2*	−0.639
14	Solexa-9081-91	−0.827	36	HS_221	−0.626
15	HS_232	−0.805	37	HS_147	−0.626
16	HS_116	−0.790	38	HS_284.1	−0.622
17	HS_2	−0.783	39	hsa-miR-671:9.1	−0.608
18	HS_188	−0.770	40	hsa-miR-665	−0.600
19	hsa-miR-566	−0.768	41	HS_17	−0.598
20	HS_135	−0.766	42	HS_38.1	−0.592
21	hsa-miR-1183	−0.749	43	hsa-miR-560:9.1	−0.589
22	HS_276.1	−0.741	​	​	​

The asterisk (*) indicates the passenger strand of the microRNA.

**TABLE 4:** Upregulated miRNAs in the recurrence-free group vs. the recurrence group.

NO	miRNA	Log2FC	NO	miRNA	Log2FC
1	hsa-miR-487b	1.360	25	hsa-miR-323-3p	0.717
2	hsa-miR-329	1.172	26	hsa-miR-342-3p	0.717
3	hsa-miR-337-3p	1.105	27	hsa-miR-374b	0.715
4	hsa-miR-335*	1.091	28	hsa-let-7b*	0.714
5	hsa-miR-410	1.083	29	hsa-miR-379*	0.713
6	hsa-miR-99a	1.046	30	hsa-miR-139-5p	0.707
7	hsa-miR-409-3p	1.018	31	hsa-miR-148b	0.694
8	hsa-miR-432	0.933	32	hsa-miR-128	0.686
9	hsa-miR-134	0.920	33	hsa-miR-154	0.664
10	hsa-miR-664	0.904	34	hsa-miR-132	0.663
11	hsa-miR-411	0.893	35	hsa-miR-99a*	0.663
12	hsa-miR-379	0.892	36	hsa-miR-574-3p	0.656
13	hsa-miR-376c	0.887	37	hsa-miR-100	0.651
14	hsa-miR-495	0.858	38	hsa-miR-889	0.640
15	hsa-miR-127-3p	0.843	39	hsa-miR-218	0.630
16	hsa-miR-369-3p	0.839	40	hsa-miR-656	0.628
17	hsa-miR-99b	0.821	41	hsa-miR-370	0.625
18	hsa-miR-10b	0.809	42	hsa-miR-376a	0.621
19	hsa-miR-154*	0.807	43	hsa-miR-377	0.621
20	hsa-miR-655	0.801	44	hsa-let-7a*	0.615
21	hsa-miR-487a	0.761	45	hsa-miR-10a	0.612
22	hsa-miR-549	0.757	46	hsa-miR-936	0.583
23	hsa-miR-431	0.739	47	hsa-miR-382	0.581
24	hsa-miR-125b	0.730	​	​	​

The asterisk (*) indicates the passenger strand of the microRNA.

**TABLE 5:** Relative expression levels of miR-495 in different tissues.

Tissue source	*p*-val	Fold change
Gastrointestinal stromal tumor	0.009	2.000
Synovial sarcoma	0.052	1.610
Fibromatosis	0.193	1.380
Malignant fibrous histiocytoma	0.143	0.748
Osteosarcoma	0.000	0.374
Malignant peripheral nerve sheath tumor	0.000	1.660
Ewings sarcoma	0.383	1.180
Schwannoma	0.268	1.180
Solitary fibrous tumor	0.674	1.110
Neurofibroma	0.004	0.647
Leiomyosarcoma	0.000	0.610
Embryonal rhabdomyosarcoma	0.000	4.180
Alveolar rhabdomyosarcoma	0.000	3.640
Normal smooth muscle	0.069	0.521
Normal skeletal muscle	0.026	0.393
Dedifferentiated liposarcoma	0.006	0.379
Well-differentiated liposarcoma	0.000	0.318
Angiosarcoma	0.005	0.256
Chondrosarcoma	0.002	3.410
Normal bone	0.307	1.460
Histiocytoma	0.325	1.360
Spindle cell sarcoma	0.906	1.060
Leiomyoma	0.712	0.885
Epithelioid sarcoma	0.128	0.558
Fibrosarcoma	0.001	0.380

### Expression of miR-495 in osteosarcoma and its correlation with patient prognosis

qRT-PCR analysis showed that miR-495 expression was significantly lower in osteosarcoma cells than in normal osteoblastic cells hFOB1.19 (*p* < 0.05). Among the osteosarcoma cell lines examined, miR-495 expression followed the order: U2OS > MG63 > SaOS2 > HOS > 143B (Figure 1E).

Consistently, miR-495 expression was significantly reduced in 60 pairs of osteosarcoma tissues compared with adjacent normal tissues (Figure 1F). Kaplan-Meier survival analysis revealed that osteosarcoma patients with low miR-495 expression had a median survival time of 42.22 months (95% CI: 32.38–52.06), while those with high miR-495 expression had a median survival time of 54.22 months (95% CI: 49.01–59.43). A Log-Rank test (*p* = 0.024) indicated that miR-495 expression levels were positively correlated with patient cumulative survival rate (Figure 1G).

### miR-495 inhibits the proliferation, invasion, and migration of osteosarcoma cells and promotes tumor cell apoptosis

**FIGURE 2:**
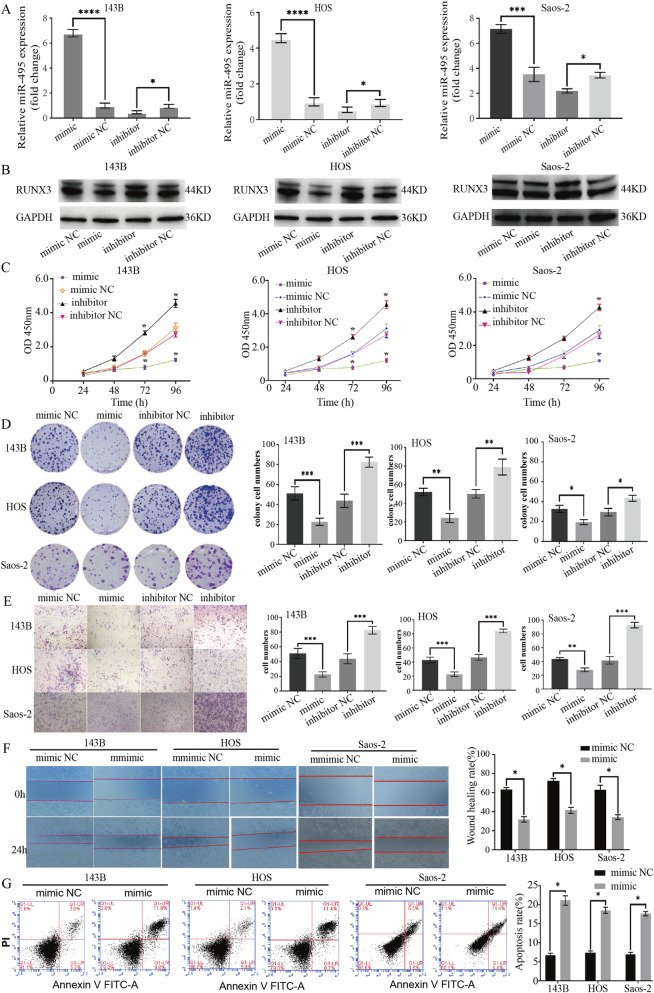
miR-495 inhibits proliferation, invasion, and migration while promoting apoptosis in osteosarcoma cells. **(A)** qRT-PCR analysis of miR-495 expression in osteosarcoma 143B, HOS and Saos-2 cells after transfection with miR-495 mimic, miR-495 negative control (miR-495 NC), miR-495 inhibitor, or miR-495 inhibitor negative control (miR-495 inhibitor NC). **(B)** Western blotting was performed to compare the expression changes of RUNX3 in osteosarcoma 143B, HOS and Saos-2 cells transfected with miR-495 mimic, miR-495 NC, miR-495 inhibitor, or miR-495 inhibitor NC. **(C)** CCK-8 assay was used to detect changes in the proliferative capacity of osteosarcoma 143B; HOS and Saos-2 cells after transfection with miR-495 mimic, miR-495 NC, miR-495 inhibitor, and miR-495 inhibitor NC. **(D)** Changes in the colony-forming ability of osteosarcoma 143B, HOS and Saos-2 cells after transfection with miR-495 mimic, miR-495 NC, miR-495 inhibitor, and miR-495 inhibitor NC. **(E)** Transwell invasion assay was performed to compare the invasive capacity of osteosarcoma 143B, HOS and Saos-2 cells transfected with miR-495 mimic, miR-495 NC, miR-495 inhibitor, and miR-495 inhibitor NC. **(F)** Wound healing assay was used to compare the migratory capacity of osteosarcoma 143B, HOS and Saos-2 cells transfected with miR-495 mimic, miR-495 NC, miR-495 inhibitor, and miR-495 inhibitor NC. **(G)** Apoptosis of osteosarcoma 143B, HOS and Saos-2 cells after transfection with miR-495 mimic, miR-495 NC, miR-495 inhibitor, and miR-495 inhibitor NC. All quantitative data are presented as mean ± SD from three independent biological replicates. Comparisons between multiple groups were analyzed using one-way ANOVA followed by appropriate *post hoc* tests. p < 0.05.

Transfection with miR-495 mimics significantly increased miR-495 expression in 143B, HOS, and SaOS2 cells, while the inhibitor group showed lower levels compared to the inhibitor NC group (Figure 2A). Increased miR-495 expression was accompanied by a marked reduction in RUNX3 expression, while miR-495 inhibition resulted in RUNX3 upregulation (Figure 2B).

Functional assays demonstrated that miR-495 overexpression significantly suppressed cell proliferation, colony formation, invasion, and migration in all three osteosarcoma cell lines, as assessed by CCK-8, colony formation, Transwell invasion, and wound healing assays, respectively (Figures 2C–F). In contrast, miR-495 overexpression markedly increased apoptotic rates compared with control cells, as determined by flow cytometry (Figure 2G).

### RUNX3 is a target gene of miR-495

**FIGURE 3:**
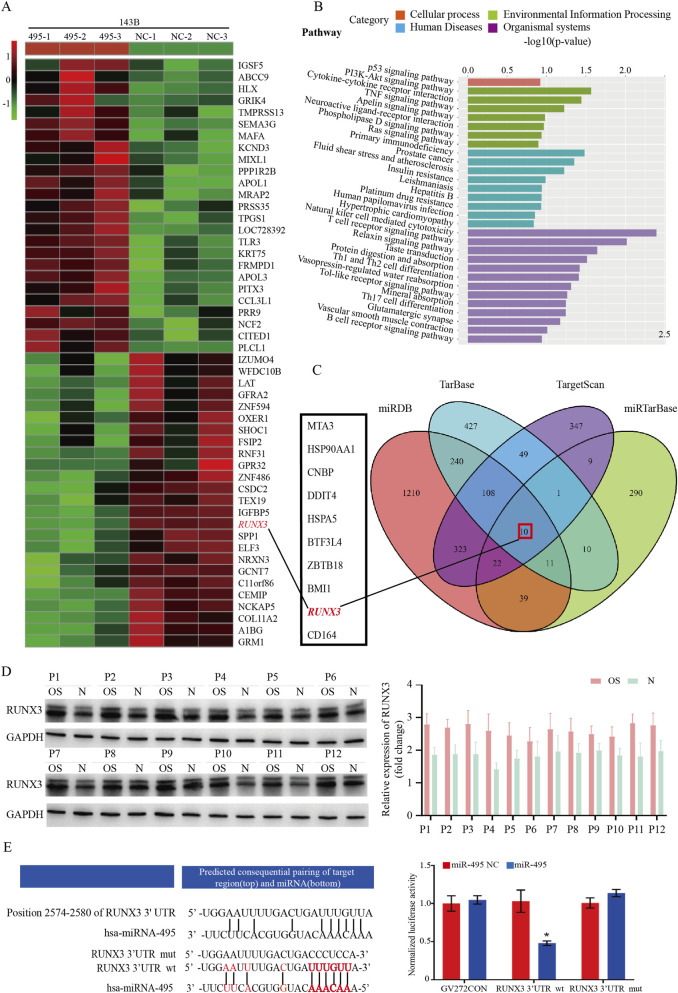
RUNX3 is a direct target gene of miR-495. **(A)** Heatmap of differentially expressed genes from RNA sequencing of osteosarcoma 143B-NC and 143B-495 cells, showing only the top 50 genes with the most significant differences. **(B)** KEGG pathway enrichment analysis of differentially expressed genes from RNA sequencing of osteosarcoma 143B-NC and 143B-495 cells. **(C)** miR-495 target genes were predicted by miRDB, TarBase, TargetScan, and miRTarBase databases. Gene intersections were identified, and a Venn diagram was generated. **(D)** Protein expression levels of RUNX3 in 12 pairs of osteosarcoma tissues (OS) and adjacent normal tissues (N) were analyzed. RUNX3 expression was higher in OS tissues than in N tissues. **(E)** Luciferase reporter assay for miR-495 targeting the 3′untranslated region (3′UTR) of RUNX3. Data are shown as mean ± SD from three independent experiments. Statistical significance was assessed using Student’s t-test or one-way ANOVA as appropriate. p < 0.05.

RNA sequencing (RNA-seq) analysis of 143B cells transfected with miR-495 mimic versus NC identified 126 upregulated and 45 downregulated genes (Figure 3A). KEGG pathway enrichment analysis of differentially expressed genes was performed using the EnrichKEGG function in the R package clusterprofiler (Yu et al., 2012), identifying significant alterations in multiple cancer-related signaling pathways (Figure 3B). Target genes of miR-495 predicted by miRDB, TarBase, TargetScan, and miRTarBase were intersected and visualized by a Venn diagram, with common predicted targets including DDIT4, MTA3, HSP90AA1, CNBP, BMI1, ZBTB18, BTF3L4, RUNX3, and CD164. Integration of RNA-seq and bioinformatics predictions identified RUNX3 as a target gene of miR-495 (Figure 3C). Western blot analysis further confirmed that RUNX3 protein expression was significantly higher in 12 pairs of osteosarcoma tissues (OS) than in adjacent normal tissues (N) (Figure 3D).

Dual-luciferase reporter assays showed that co-transfection of RUNX3 mRNA 3′UTR wild-type (wt) and miR-495 mimic in 293T cells resulted in a relative luciferase activity of 0.45 ± 0.06, significantly lower than the control group (*p* < 0.05), indicating direct binding and luciferase activity inhibition. In contrast, co-transfection of RUNX3 mRNA 3′UTR mutant (mut) and miR-495 mimic showed no significant difference in luciferase activity compared to the control (*p* > 0.05), confirming failed binding. These results demonstrate that miR-495 directly recognizes and binds to the RUNX3 mRNA 3′UTR, suppressing RUNX3 mRNA translation and protein expression, thus validating RUNX3 as a target gene of miR-495 (Figure 3E).

### miR-495 directly targets and suppresses RUNX3 expression

**FIGURE 4:**
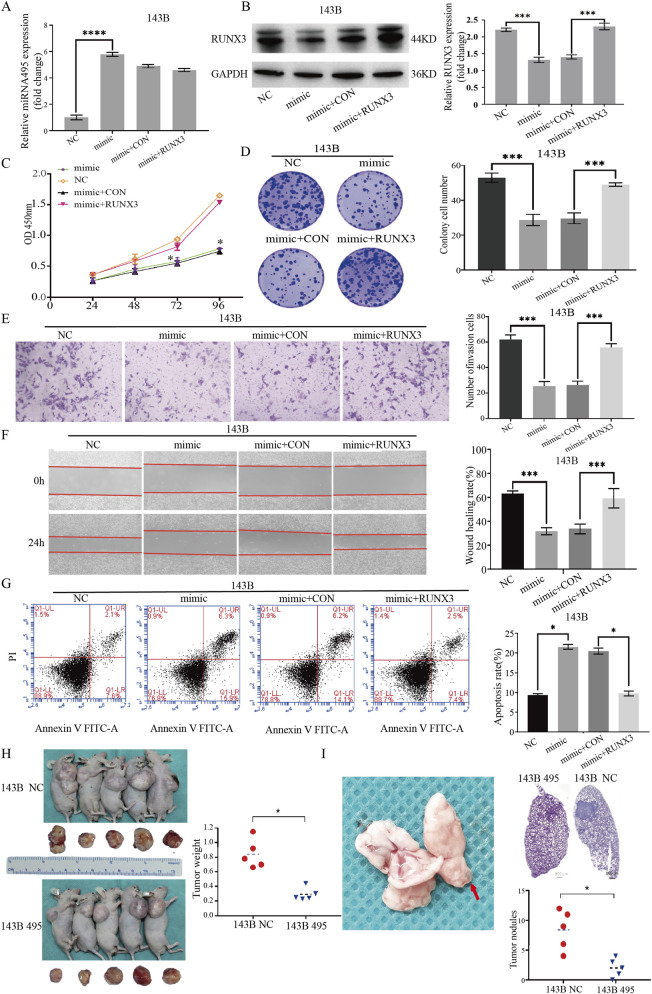
RUNX3 promotes the proliferation, invasion, and migration of osteosarcoma cells while reducing apoptosis. **(A)** Expression levels of miR-495 in osteosarcoma 143B cells transfected with miR-495 mimic, miR-495 NC, RUNX3 overexpression plasmid (RUNX3), and empty vector control (RUNX3 CON). **(B)** Protein expression levels of RUNX3 in osteosarcoma 143B cells transfected with miR-495 mimic, miR-495 NC, RUNX3, and RUNX3 CON. **(C)** Changes in the proliferative capacity of osteosarcoma 143B cells after transfection with miR-495 mimic, miR-495 NC, RUNX3, and RUNX3 CON. **(D)** Changes in the colony-forming ability of osteosarcoma 143B cells after transfection with miR-495 mimic, miR-495 NC, RUNX3, and RUNX3 CON. **(E)** Changes in the invasive capacity of osteosarcoma 143B cells after transfection with miR-495 mimic, miR-495 NC, RUNX3, and RUNX3 CON. **(F)** Changes in the migratory capacity of osteosarcoma 143B cells after transfection with miR-495 mimic, miR-495 NC, RUNX3, and RUNX3 CON. **(G)** Apoptosis of osteosarcoma 143B cells after transfection with miR-495 mimic, miR-495 NC, RUNX3, and RUNX3 CON. **(H) **Subcutaneous xenograft tumor models of osteosarcoma were established in nude mice using osteosarcoma 143B-495 and 143B-control cells, demonstrating that miR-495 inhibits osteosarcoma growth *in vivo*. **(I)** In lung metastasis animal model established via tail vein injection, the number of lung metastatic foci formed by osteosarcoma 143B-495 cells was significantly reduced compared with the osteosarcoma 143B-NC cell group. *In vitro* experiments were performed with three independent biological replicates, *in vivo* experiments n = 5. Data are presented as mean ± SD. Statistical comparisons among multiple groups were conducted using one-way ANOVA with *post hoc* testing. *p* < 0.05.

qRT-PCR result showed that miR-495 levels in cells transfected with miR-495 mimic were significantly higher than those in the NC group, while miR-495 levels remained unchanged in the co-transfection of miR-495 and RUNX3 (Figure 4A, *p* < 0.05).

WB analysis revealed that RUNX3 protein expression was significantly reduced in the miR-495 mimic group, with a statistically significant difference compared to the NC group (*p* < 0.05). Co-transfection of miR-495 and the RUNX3 overexpression vector restored RUNX3 expression levels compared to the mimic + CON group (Figure 4B), indicating that RUNX3 transfection reversed the inhibitory effect of miR-495 on RUNX3.

### RUNX3 promotes the proliferation, invasion, and migration of osteosarcoma cells while suppressing apoptosis

CCK-8 assay results showed that the restoration of RUNX3 expression reversed the inhibitory effect of miR - 495 on the proliferative capacity of osteosarcoma 143B cells and promoted cell viability (Figure 4C).

Colony formation assays indicated that increased miR-495 levels suppressed the colony forming ability of osteosarcoma cells, while the simultaneous overexpression of RUNX3 restored colony formation (Figure 4D).

Regarding the invasion ability (Figure 4E), the number of 143B osteosarcoma cells penetrating Matrigel in the mimic group was 25 ± 3.3, which was significantly lower than that in the NC control group (62 ± 5.1, *p* < 0.05). Co-transfection with mimic and RUNX3 increased the number of invasive cells to 55 ± 3.1, compared to 24 ± 2.1 in the mimic + CON group (*p* < 0.05).

Wound healing assays showed that the scratch repair rate in the mimic group was significantly lower than that in the NC control group after 24 h (Figure 4F, *p* < 0.05). No significant difference in the scratch repair rate was observed between the mimic group and the mimic + CON group (*p* > 0.05), but the mimic + RUNX3 group exhibited a significantly higher repair rate than the mimic + CON group (*p* < 0.05).

Flow cytometry analysis of apoptosis in osteosarcoma 143B cells revealed that the apoptosis rate was 9.2% ± 0.15% in the miR - 495 NC group, while the overexpression of miR - 495 in the mimic group significantly increased apoptosis to 21.5% ± 0.19% (Figure 4G, *p* < 0.05), indicating that miR-495 promotes apoptosis. Co-transfection with miR - 495 mimic and RUNX3 reduced the apoptosis rate to 9.8% ± 0.17%, whereas co - transfection with miR-495 mimic and control plasmid CON showed no significant change (20.7% ± 0.16%, *p* > 0.05). Similar results were observed when comparing the mimic group and the mimic + CON group (*p* > 0.05). These findings suggest that upregulating RUNX3 protein expression counteracts the pro - apoptotic effect of miR-495 in osteosarcoma 143B cells.

### miR-495 suppresses the growth of murine xenograft tumors and lung metastasis

Subcutaneous xenograft models of osteosarcoma were established using 143B-495 and 143B-NC cells in nude mice. Results showed that miR-495 significantly inhibited tumor growth *in vivo* (Figure 4H). Additionally, lung metastasis models established via tail vein injection confirmed that the number of lung metastatic nodules in the 143B-495 cell group was significantly reduced compared to the 143B-NC group 4 weeks after injection (Figure 4I, *p* < 0.05). These findings indicate that miR-495 suppresses both the growth and metastasis of osteosarcoma cells in animal models.

### miR-495 exerts its function through the RUNX3/PI3K/Akt pathway

WB analysis was performed to detect changes in PI3K/Akt pathway-related molecules in osteosarcoma 143B cells transfected with: ① miR-495 NC (NC), ② miR-495 mimic (mimic), ③ miR-495 mimic + RUNX3 CON (mimic + CON), or ④ miR-495 mimic + RUNX3 (mimic + RUNX3). High miR-495 expression significantly downregulated the levels of PI3K, Akt, p-Akt (Ser473), mTOR, p70S6K, and Bcl-2, inhibiting PI3K/Akt pathway activity. Concurrently, the expressions of Bax, cleaved-caspase-3 (c-casp3), and cleaved-caspase-9 (c-casp9) increased compared to the control group, promoting tumor cell apoptosis.

**FIGURE 5:**
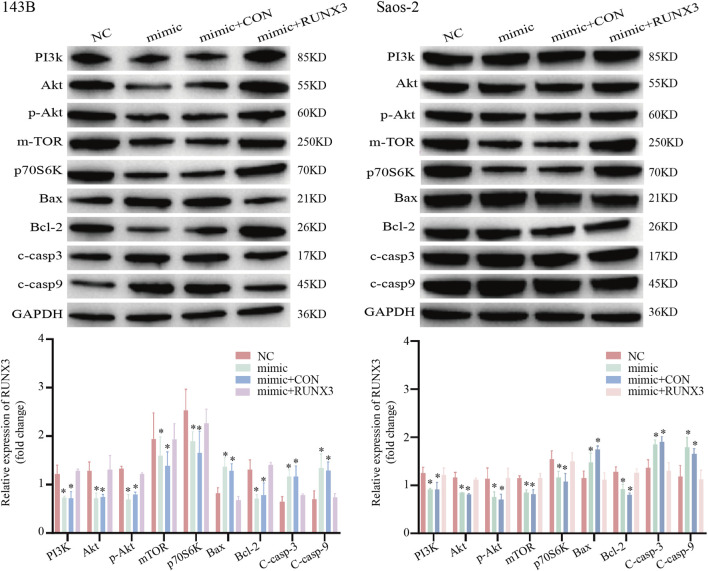
miR-495 exerts its function through the RUNX3/PI3K/Akt pathway. Western blot analysis of changes in key molecules of the PI3K/Akt pathway and apoptosis-related proteins in osteosarcoma 143B and Saos-2 cells co-transfected with miR-495 mimic and RUNX3 overexpression plasmid. Representative blots from three independent experiments are shown. Quantitative data are expressed as mean ± SD. Statistical significance was determined using one-way ANOVA followed by *post hoc* testing. *p* < 0.05.

Co-transfection with miR-495 and RUNX3 revealed that restoration of RUNX3 protein expression rescued the levels of key molecules including PI3K, Akt, p-Akt(Ser473), mTOR, p70S6K, and Bcl-2. Correspondingly, the expressions of Bax, c-casp3, and c-casp9 were partially suppressed, alleviating the inhibition of the PI3K/Akt pathway (Figure 5). These results indicate that miR-495 overexpression suppresses the levels of PI3K, Akt, p-Akt(Ser473), mTOR, p70S6K, and Bcl-2, which are rescued by increased RUNX3 protein expression, with opposite effects observed for Bax, c-casp3, and c-casp9. No significant differences in these molecular changes were detected between the miR-495 mimic group and the miR-495 mimic + CON group (*p* > 0.05). Collectively, these findings demonstrate that miR-495 inhibits PI3K/Akt pathway activity by targeting RUNX3.

## Discussion

Osteosarcoma, the most common primary malignant bone tumor in adolescents, is characterized by abnormal osteogenesis by tumor cells. Although neoadjuvant chemotherapy combined with limb-sparing surgery has improved the 5-year survival rate, recurrence and metastasis remain critical therapeutic bottlenecks that severely compromise patient prognosis (Cole et al., 2022; Song et al., 2019). Osteosarcoma exhibits remarkable heterogeneity and diverse pathological subtypes, with its cellular origin and genetic drivers remaining incompletely understood. Current evidence suggests that mutations in osteoblasts during differentiation may represent a key event in osteosarcoma development, but the specific molecular mechanisms require further exploration.

The advancement of gene microarray and high-throughput sequencing technologies have provided new tools for studying tumor molecular mechanisms. Notably, the 2024 Nobel Prize in Physiology or Medicine’s recognition of microRNA research highlights the pivotal role of these molecules in gene regulatory networks. This study focused on the screening and functional characterization of osteosarcoma-associated miRNAs, aiming to decipher their regulatory mechanisms in tumor progression and provide a theoretical basis for developing novel diagnostic and therapeutic strategies.

Through integrated bioinformatics analyses and validation in clinical samples, this study confirmed that miR-495 expression was reduced in osteosarcoma tissues and was positively associated with patient survival. These findings establish the potential value of miR-495 as a prognostic biomarker for osteosarcoma. Functional studies further demonstrated that miR-495 overexpression inhibits the malignant phenotype of osteosarcoma through multiple mechanisms: it not only significantly reduces cell proliferation, colony formation, migration, and invasion but also induces apoptosis and suppresses xenograft tumor growth and lung metastasis *in vivo*. Mechanistic investigations revealed that miR-495 suppresses PI3K/Akt signaling pathway activity by targeting the RUNX3 gene, thereby blocking tumor progression. This discovery establishes a novel miR-495/RUNX3/PI3K/Akt regulatory axis, providing new insights into the pathogenesis of osteosarcoma.

The innovations of this study are reflected in the following aspects compared with previously reported osteosarcoma-related miRNAs: First, although molecules such as miR-144-3p, miR-125b, and miR-598 have been confirmed to be involved in osteosarcoma regulation, their mechanisms of action are mostly limited to single pathways or targets (Jiang et al., 2023; Xiao et al., 2019; Liu et al., 2017). This study reveals the miR-495/RUNX3/PI3K/Akt regulatory network, which not only integrates the cascading effects of post-transcriptional regulation and signaling pathway activation but also verifies its multidimensional inhibitory effects on tumor growth and metastasis through *in vitro* and *in vivo* experiments. Second, although studies have reported that molecules such as miR-22 exert tumor-suppressive effects through the PI3K/Akt pathway (Chen et al., 2023), this study is the first to establish RUNX3 as a direct target gene of miR-495 and clarify the specific role of this regulatory axis in osteosarcoma. This systematic analysis of upstream regulatory molecules and downstream pathways expands the depth of molecular mechanism research in osteosarcoma.

RUNX3, a member of the runt domain transcription factor family, has drawn extensive attention due to its dual roles in different cancers (Otani et al., 2022; Bledsoe et al., 2014; Omori et al., 2023). Our study revealed that RUNX3 is highly expressed and exhibits pro-tumorigenic activity in osteosarcoma tissues. miR-495 was found to inhibit RUNX3 expression by targeting its 3′UTR region, thereby blocking the PI3K/Akt pathway. This discovery not only confirms the oncogenic role of RUNX3 in osteosarcoma but also unveils a novel mechanism by which miR-495 suppresses RUNX3 expression through post-transcriptional regulation. By integrating transcriptomic sequencing and miRNA target prediction, we precisely identified RUNX3 as a functional target of miR-495. This multi-dimensional validation strategy enhances the reliability of our conclusions.

At the signaling pathway level, abnormal activation of the PI3K/Akt pathway has been confirmed as a key driver of osteosarcoma progression (Chen et al., 2021; Wang Y. et al., 2024; Zhang et al., 2024). Through KEGG pathway enrichment analysis and Western blot experiments, this study confirmed that miR-495 significantly downregulates the expression levels of PI3K, Akt, and its phosphorylated form (p-Akt), while regulating the expression of apoptosis-related proteins (Bax, cleaved-caspase-3 [c-casp3], cleaved-caspase-9 [c-casp9], and Bcl-2). Notably, RUNX3 overexpression reversed the inhibitory effect of miR-495 on the PI3K/Akt pathway, and this Rescue experiment directly demonstrated the central role of the miR-495/RUNX3 axis in regulating the PI3K/Akt pathway. This chain-of-interaction analysis of molecular interactions provides a clear theoretical basis for targeted interventions.

This study systematically elucidates the tumor-suppressive role and molecular mechanisms of miR-495 in osteosarcoma, providing new targets for osteosarcoma diagnosis, prognostic evaluation, and targeted therapy (such as miR-495 mimics or RUNX3 inhibitors). However, the study has limitations: ① miR-495 may have other target genes (such as DDIT4 and CNBP), which require further validation; ② the role of miR-495 in chemoresistance remains unclear; ③ whether miR-495 represents an independent prognostic factor and its mechanism in clinical samples needs verification with a larger sample size. Future research could focus on the synergistic effects of miR-495 combined with PI3K inhibitors or explore its potential clinical application as an exosomal biomarker, providing a theoretical basis for precision medicine in osteosarcoma.

## Conclusion

**FIGURE 6:**
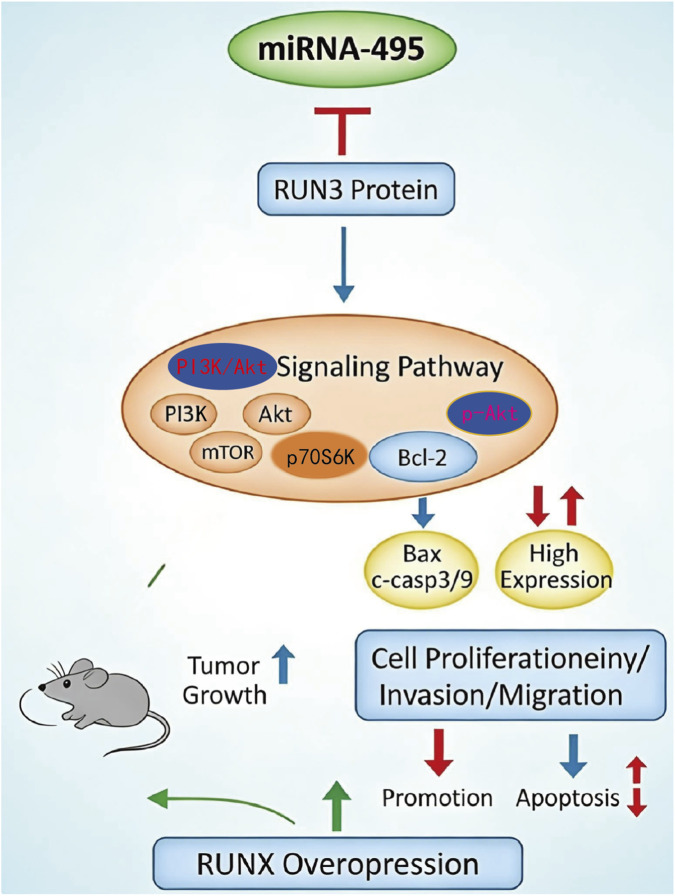
Schematic diagram of the mechanism by which miR-495 inhibits osteosarcoma progression via regulating the RUNX3/PI3K-Akt pathway.

Through multi-omics analysis and functional validation, this study demonstrates that miR-495 suppresses osteosarcoma progression by targeting RUNX3 and modulating PI3K/Akt signaling (Figure 6). These findings expand current understanding of the molecular mechanisms underlying osteosarcoma and suggest that the miR-495/RUNX3 axis may represent a potential target for future diagnostic and therapeutic strategies. Further investigation is warranted to evaluate the clinical applicability of miR-495-based interventions.

## Data Availability

The datasets used and analyzed in this study are available from the corresponding authors upon reasonable request. The RNA-seq data generated in this study have been deposited in the National Genomics Data Center (accession number: HRA016847).
